# Analysis of possible risk factors for the severity of paediatric obstructive sleep apnoea syndrome

**DOI:** 10.1007/s00405-023-08237-w

**Published:** 2023-09-27

**Authors:** Lea Dékány, Viktória Molnár, András Molnár, András Bikov, Zsófia Lázár, Orsolya Bárdos-Csenteri, Pálma Benedek

**Affiliations:** 1 Department of Otolaryngology and Head and Neck Surgery, Semmelweis University, Budapest, Hungary; 2 Wythenshawe Hospital, Manchester University NHS Foundation Trust, Manchester, UK; 3 Department of Pulmonology, Semmelweis University, Budapest, Hungary; 4 Gottsegen György Hungarian Institute of Cardiology, Budapest, Hungary; 5 Sleep Laboratory and Sleep Surgery Unit, Heim Pál National Paediatric Institute, Budapest, Hungary

**Keywords:** Obstructive sleep apnoea, Obesity, Asthma, Sex, Risk factors, Paediatric

## Abstract

**Purpose:**

This study aimed to determine the effect of body mass index (BMI) percentile, asthma, sex, and age on the paediatric obstructive sleep apnoea (OSA) severity. Furthermore, to determine the possible predictive role of the BMI percentile and age in severe OSA.

**Methods:**

This retrospective study included 921 children aged 2–18 years diagnosed with OSA by polysomnography. Analysis of Covariance (ANCOVA), Spearman’s correlation, Receiver Operating Characteristics (ROC) analyses were performed and area under the curve (AUC) was determined.

**Results:**

We observed a significant association between a higher BMI percentile and the severity of OSA (p < 0.001, ρ = 0.15). The correlation also was significant under (*p* = 0.007, ρ = 0.11) and over 7 (*p* = 0.0002, ρ = 0.23) years of age. There was no association between the severity of OSA and the presence of asthma (*p* = 0.9) or sex (*p* = 0.891), respectively. Age was significantly related to OSA severity (*p* = 0.01, *ρ* = 0.08). Although both the BMI percentile (0.59 AUC [0.54–0.65]) and age (0.58 AUC [0.52–0.63]) predicted severe OSA, according to the sensitivity and specificity values of the ROC curve, the association presents a slight clinical relevance.

**Conclusions:**

OSA severity is determined by the BMI percentile and age in children; however, these factors are unsuitable for predicting severe OSA in clinical practice. Based on our results, obesity is also a significant risk factor for OSA in younger children. Our study highlights that older, overweight, and obese children have a higher risk for severe OSA.

## Introduction

Sleep-disordered breathing (SDB) is a common condition in the paediatric population. The most severe form of obstructive SDB is obstructive sleep apnoea (OSA) [1]. OSA is a breathing disorder during sleep that involves periods of partial or complete obstruction of the upper airways, disrupting normal sleep patterns [2]. The prevalence of paediatric OSA ranges from 1.2% to 5.7% [3]. However, it affects 5.7–56% of obese children [4]. This condition is the most common among children aged 2–8 years, as the size of the lymphoid tissues of the upper airways is the largest during these years [5].

The two main established risk factors for paediatric OSA are adenotonsillar hypertrophy and obesity. However, the contribution of adenoid size declines in adolescence [5, 6]. Nowadays, excess weight in childhood is one of the most critical public health issues. The prevalence of obesity increases with age; however, under 5 years of age, the problem is also exceedingly relevant [7]. Age may be an essential factor in improving the effect of obesity on the severity of OSA. The effect of Body Mass Index (BMI) on OSA severity is more considerable in older age groups [8]. Since obesity in younger children is also a serious health condition, further research is required on the association between obesity and OSA in younger and older children. OSA in underweight children has not yet been thoroughly studied, although according to the literature, the underweight status may increase the risk of paediatric OSA [9].

Bronchial asthma, gastroesophageal reflux disease (GERD), and allergic rhinitis are also commonly considered risk factors [10]. The relationship between asthma and OSA in children is supported by several lines of evidence, and depends on asthma control and the consequential need for systemic corticosteroids [11]. Furthermore, inflammation of the united airways could contribute to the link [12, 13].

Paediatric OSA can cause severe disease-associated complications regarding neurobehavioral, metabolic and cardiovascular consequences, excessive daytime sleepiness and somatic growth impairment [14]. Early diagnosis and management are critical to reducing OSA complications, as well as the development of easily applicable screening methods.

In this study, we aimed to analyse the impact of age, sex, asthma, and different body weight statuses on the severity of OSA in a large cohort of paediatric patients. Furthermore, to investigate whether age and BMI percentile alone are suitable for predicting severe OSA.

## Materials and methods

### Participants and inclusion criteria

**Fig. 1:**
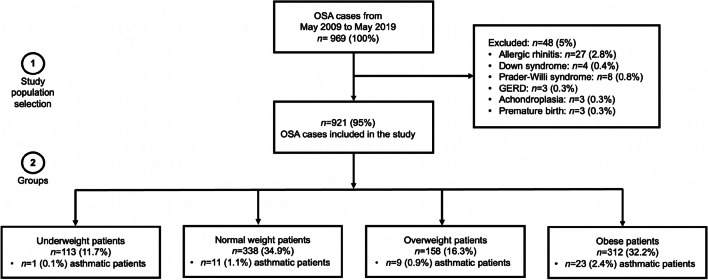
Study population selection and the four groups created according to the BMI percentiles

Our study is based on retrospective data analysis of paediatric OSA patients examined between May 2009 and May 2019. Each case was presented and diagnosed at the Paediatric Sleep Laboratory of Heim Pál National Paediatric Institute, Budapest, Hungary. The two inclusion criteria were (1) aged 2–18 years; (2) the apnoea–hypopnea index (AHI ≥ 1) according to the overnight polysomnography results. Exclusion criteria included allergic rhinitis, Down syndrome, Prader–Willi syndrome, GERD, achondroplasia, and premature birth, concerning that these conditions influence OSA [5, 10, 15]. In total, 921 children (573 male, median age was 5 years [Interquartile range (IQR): 3–7]) met the initial inclusion criteria for the study. Children were divided into four groups according to the BMI percentile as follows: underweight (< 5; *n* = 113), normal weight (5–84; *n* = 338), overweight (85–94; *n* = 158), and obese (≥ 95; *n* = 312) [16]. The BMI percentiles were calculated by Centres for Disease Control and Prevention (CDC) calculator based on CDC growth charts (https://www.cdc.gov/healthyweight/bmi/calculator.html). Self-reported asthma was only registered if patients used an asthma medication at the time of the examination [17]. Overall, there was one asthmatic patient in the underweight, 11 in the normal weight, 9 in the overweight and 23 in the obese group. The study population selection is summarised in Fig. 1. Furthermore, two age subgroups were created (i.e., under and over 7 years of age), as in previous studies [8, 18].

The study protocol of retrospective data analysis was approved by the Ethics Committee of the Heim Pál National Paediatric Institute, Budapest (number of the Ethical Approval: KUT-15/2021) and followed the Declaration of Helsinki. Informed consent was not required due to the retrospective design.

### Patient examinations and polysomnography

A medical history, including comorbidities, was taken, and physical examinations, including height and weight measurements, were performed. The diagnosis of OSA was based on overnight polysomnography using a Somnomedics Somnoscreen plus device (Somnomedics, Randersacker, Germany) [2, 19]. Apnoeas and hypopneas were analysed, and AHI was determined as the total number of apnoeas and hypopneas per hour of sleep. The desaturation index (DI), i.e., the number of periods with desaturation per hour (i.e., a minimum 4% decrease in oxygen saturation), was also calculated [20, 21]. In addition, the minimum oxygen saturation level (min. SpO_2_) was also obtained. The diagnostic criteria of the American Academy of Sleep Medicine defined paediatric OSA diagnosis as AHI ≥ 1 [22]. In the present study, OSA was classified as mild (1 ≤ AHI < 5), moderate (5 ≤ AHI < 10), and severe (AHI ≥ 10) [23].

### Statistical analysis

Statistical analysis was performed using SPSS Statistics 28.0.1.0 (142) (IBM Corp., Armonk, N.Y., USA) and JASP 0.14.1 software (University of Amsterdam, Amsterdam, Netherlands). Continuous variables were presented as median and interquartile range (IQR), while categorical variables were expressed as percentages. The normality of the continuous data was determined by the Kolmogorov–Smirnov test, which indicated a not normal distribution for AHI, BMI and age. Consequently, the Kruskal–Wallis, Dunn’s post hoc and Chi-square tests were used to compare data between subgroups. The relationships between BMI percentile, age and AHI were analysed with Spearman’s correlation in the whole study population and the pre-specified age groups. Furthermore, the correlations between OSA severity and BMI percentile, age, sex and asthma were analysed with a non-parametric analysis of covariance (ANCOVA). In addition, receiver operating characteristic (ROC) curves were computed, and area under the curve (AUC) values were calculated to investigate whether age and BMI percentile predict severe OSA. A *p*-value < 0.05 was defined as statistically significant in all cases.

## Results

### Patients’ characteristics

The participants’ characteristics who met the study inclusion criteria (see Fig. 1) are summarised in Table 1.

**Table 1:** Patients’ characteristics by BMI percentile categories

	Underweight patients (*n* = 113)	Normal weight patients (*n* = 338)	Overweight patients (*n* = 158)	Obese patients (*n* = 312)	*p-* value
Demographic/anthropometric values					
Age, median (IQR), years	4 (3–5)^e^^,^^f^^,^^d^	4 (3–6)^e^^,^^c^^,^^a^	5 (4–9)^f^^,^^c^	6 (4–8)^d^^,^^a^	< 0.001^*^
Age, under and over 7 years of age					< 0.001^*^
< 7, *n* (%), years	102 (90.3)	259 (76.6)	95 (60.1)	197 (63.1)	
≥ 7, *n* (%), years	11 (9.7)	79 (23.4)	63 (39.9)	115 (36.9)	
Sex, *n* (%)					0.533
Male	71 (62.8)	200 (59.2)	101 (63.9)	201 (64.4)	
Female	42 (37.2)	138 (40.8)	57 (36.1)	111 (35.6)	
Height, median (IQR), cm	104 (98–110)^f^^,^^d^	105 (95–119)^c^^,^^a^	113 (98–136)^f^^,^^c^	119 (105–135)^d^^,^^a^	< 0.001^*^
Weight, median (IQR), kg	14 (12–16)^e^^,^^f^^,^^d^	17 (14–22)^e^^,^^c^^,^^a^	23 (17–35)^f^^,^^c^^,^^b^	30 (22–45)^d^^,^^a^^,^^b^	< 0.001^*^
BMI percentile, median (IQR), kg/m^2^	13.3 (12.5–13.6)^e^^,^^f^^,^^d^	15.6 (14.8–16.6)^e^^,^^c^^,^^a^	18 (17.5–19.4)^f^^,^^c^^,^^b^	22 (19.7–25.5)^d^^,^^a^^,^^b^	< 0.001^*^
Sleep test parameters					
AHI, median (IQR), event/hour	2.4 (1.5–5.7)	2.2 (1.4–4.3)^c^^,^^a^	2.9 (1.6–6)^c^	3.4 (1.7–7.9)^a^	< 0.001^*^
DI, median (IQR), event/hour	4 (1.9–10.5)^d^	3 (1.6–7.7)^a^	3.5 (1.6–8.9)^b^	5 (2.6–13.5)^a^^,^^b^^,^^d^	< 0.001^*^
Min. SpO_2_, median (IQR), %	85 (79–89)	86 (79–89)^a^	86 (79–90)^b^	84 (74–88)^a^^,^^b^	< 0.001^*^
OSA category, *n* (%)					0.003^*^
Mild OSA	81 (71.7)	273 (80.8)	117 (74)	217 (69.5)	
Moderate OSA	14 (12.4)	34 (10)	23 (14.6)	33 (10.6)	
Severe OSA	18 (15.9)	31 (9.2)	18 (11.4)	62 (19.9)	
Presence of asthma, *n* (%)	1 (0.9)	11 (3.3)	9 (5.7)	23 (7.4)	0.015^*^

*IQR* Interquartile range, *BMI* Body Mass Index, *AHI* Apnoea–Hypopnea Index, *DI* Desaturation Index, *OSA* Obstructive Sleep Apnoea

Continuous variables are presented as median (IQR), while categorical variables were expressed as percentages. Continuous data were analysed by non-parametric Kruskal–Wallis test and Dunn’s post hoc test, while categorical variables by Chi-square test

^*^Indicates the significant difference at *p < *0.05. Statistically significant values are highlighted with bold emphasis in the table

^a^Significant differences between normal weight and obese categories, according to Dunn’s post hoc test

^b^Significant differences between overweight and obese categories, according to Dunn’s post hoc test

^c^Significant differences between normal weight and overweight categories, according to Dunn’s post hoc test

^d^Significant differences between underweight and obese categories, according to Dunn’s post hoc test

^e^Significant differences between underweight and normal weight categories, according to Dunn’s post hoc test

^f^Significant differences between underweight and overweight categories, according to Dunn’s post hoc test

As presented in Table 1, significantly older patients were found in the overweight and obese groups (*p* < 0.001). Overall, considerably more patients were included in the children under 7 years (*n* = 653) group than those over 7 years of age (*n* = 268). Underweight and normal-weight children were dominant in the group under 7 years of age, while over 7 years, the number of overweight and obese children was higher. Statistically significant differences were observed between the height, weight, and BMI values (*p* < 0.001) of the four groups, indicating an increasing tendency in the case of each parameter with BMI. No significant difference in the prevalence of males was found between the weight categories (*p* = 0.533). Otherwise, a predominance of male (*n* = 573) participants was detected in the current study population.

As revealed in Table 1, significant differences in sleep parameters were recorded among the groups. The AHI and the DI values were significantly higher (*p* < 0.001) in the obese group, while the min. SpO_2_ was lower (*p* < 0.001). Dunn’s post hoc test revealed a significant difference between the normal weight and obese, and between the normal weight and overweight groups regarding the AHI; however, there were no correlations between the underweight and normal weight categories. Significantly more patients with severe OSA were in the obese children group than in the other three groups (*p* = 0.003). It can also be stated that significantly more asthmatic children were found in the group of obese patients (*p* = 0.015).

### The effect of BMI percentile on OSA severity

**Fig. 2:**
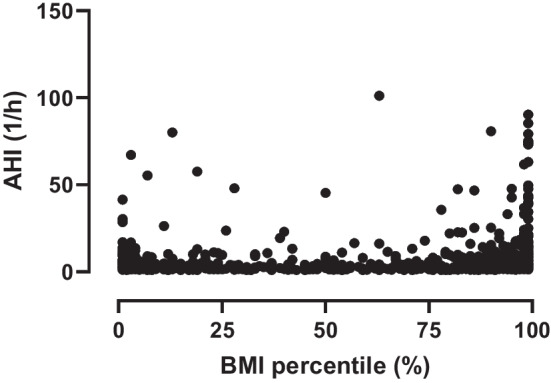
Spearman’s correlation analysis for the association between BMI percentile and OSA severity (AHI) in the whole study population. *AHI* Apnoea–Hypopnea Index, *BMI *Body Mass Index

There was a slight, although significant correlation between the BMI percentiles and OSA severity (p < 0.001, ρ = 0.15, Fig. 2). The correlation was significant in the < 7 years (*p* = 0.007, *ρ* = 0.11) and ≥ 7 years (*p < *0.001, *ρ* = 0.23) subgroups, respectively.

### The effect of age on OSA severity

**Fig. 3:**
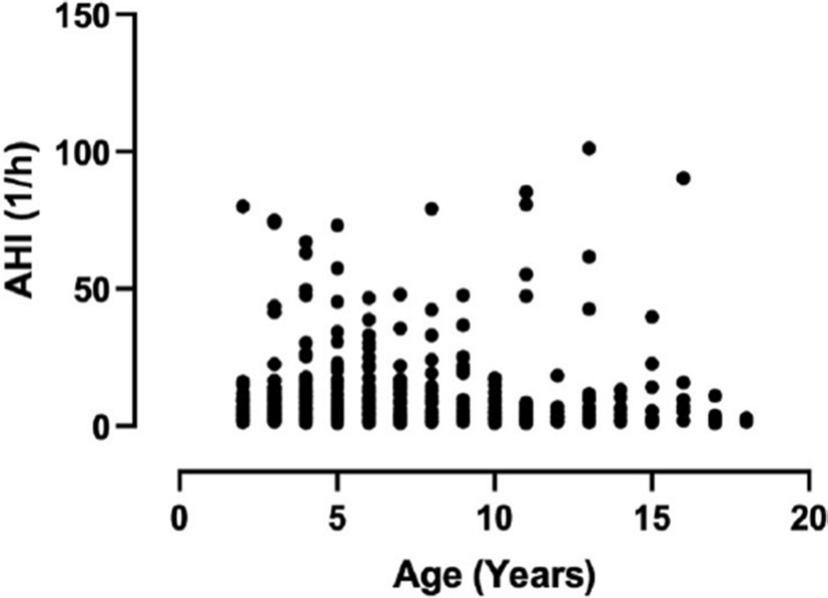
Spearman’s correlation analysis for the association between age and OSA severity (AHI) in the whole study population. *AHI* Apnoea–Hypopnea Index

In this case, a significant correlation between the AHI values and age was observed when the whole study population was analysed (*p* = 0.01, *ρ* = 0.08, Fig. 3). However, when this correlation in the four subgroups (i.e., under-, normal, overweight and obese) was analysed, a significant association was only detected in the case of the obese group (*p* = 0.008, *ρ* = 0.15).

### Possible risk factors for OSA severity

The effect of BMI, age, sex, and asthma was investigated using the non-parametric ANCOVA test. As can be observed from Table 2, the test revealed that only age was associated with OSA severity.

**Table 2:** Possible risk factors for OSA severity by non-parametric ANCOVA test

	*F* ratio	*p*-value
Age (years)	9.652	0.002^*^
Sex (male/female)	0.187	0.891
BMI percentile (%)	3.057	0.081
Presence of asthma (yes/no)	0.132	0.900

*BMI* Body Mass Index

^*^Indicates the significant difference at *p < *0.05. Statistically significant values were highlighted with bold emphasis in the table

### ROC analysis to predict severe OSA

**Fig. 4:**
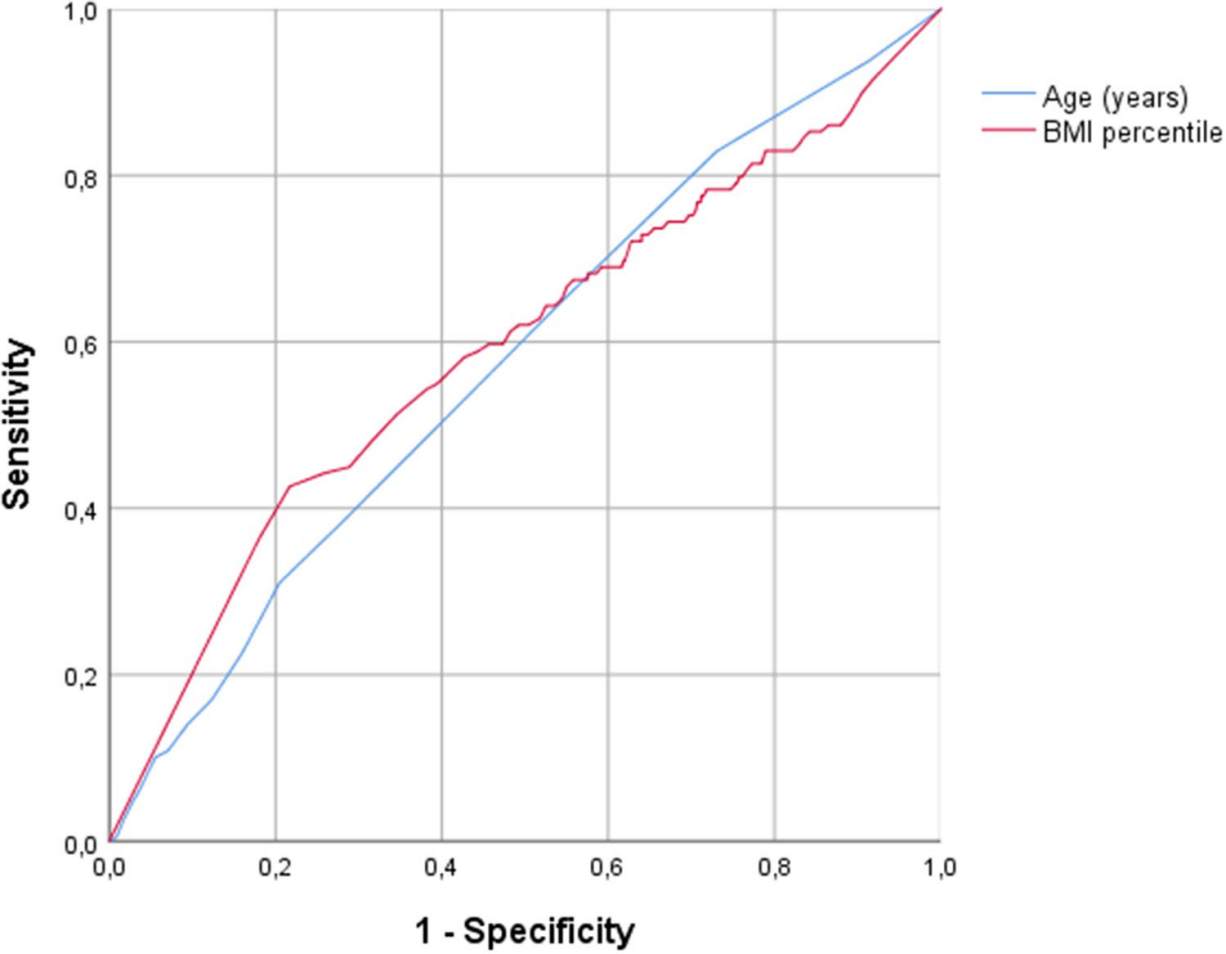
ROC curve to predict severe OSA based on age and BMI percentile values

ROC curves were drawn, and AUC values were calculated to analyse the prediction of age and BMI on OSA severity. Both age (0.58 AUC [0.52–0.63] 95% confidence interval) and BMI percentile (0.59 AUC [0.54–0.65] 95% confidence interval) significantly predicted severe OSA. However, the clinical value of this prediction is limited, as can be observed from the sensitivity and specificity values of the ROC curve (Fig. 4).

## Discussion

The present retrospective investigation concluded that the BMI percentile and the age were; however, asthma and sex were not associated with the severity of OSA in children. These results indicate that obese and older children are at an increased risk for more severe OSA. There are controversies even in the international literature about predictors of paediatric OSA. Furthermore, the differences in study design and definitions of OSA, respiratory events, or obesity make comparisons even more difficult. The effect of BMI percentile and age on OSA severity has previously been studied in several investigations. For instance, Scott et al. studied 290 children aged 2–18 years. They reported that the BMI Z-score is a poor predictor of OSA severity in young children (i.e., younger than 12 years); however, it plays a more vital role in older children (i.e., older than 12 years) [24]. Graw-Panzer et al. reported that increased BMI Z-scores were associated with increased OSA severity in children of 7 years of age or older. However, such a correlation was not detected in the case of children of 6 years or younger [8]. Our study has found a significant association between the BMI percentile and AHI in both age subgroups (i.e., under and over 7 years of age).

Our study has reported a significant correlation between the BMI percentile and AHI, using Spearman's correlation analysis. This result is in concordance with similar studies [17, 25]. Although, according to the statistical analysis, the BMI percentile did not significantly impact the severity of OSA. However, the outcome of the analysis was influenced by the fact that OSA severity was defined as categorical in this case. In Spearman’s correlation, we used AHI as a continuous variable to describe the severity of OSA. Polysomnographic parameters also represent the negative effect of BMI percentile elevation on OSA severity in our cohort. AHI and DI were significantly higher, and min. SpO2 was considerably lower in the obese subgroup than in the other weight categories. Our results and previous studies show that weight loss therapy can be a crucial intervention in overweight and obese paediatric populations [3].

Our analyses did not show a correlation between AHI values in the underweight and normal weight categories. Therefore, in our study, the underweight status did not significantly impact the OSA severity.

According to the literature, the association between paediatric asthma and OSA has been particularly investigated; however, airway inflammation and nocturnal bronchoconstriction may increase OSA risk [13, 17, 26, 27]. In the present study, self-reported asthma with medication did not increase the risk of OSA. Despite this, the results of our study do not exclude the association between the two diseases, as the number of asthmatic patients was relatively low. The international literature still has discrepancies on the association between paediatric asthma and OSA, requiring further studies to evaluate the association between the two conditions [17, 26, 27].

In addition, there were more asthmatic patients in the overweight and obese groups than in the normal weight and underweight categories in the present cohort. This may be explained by the significant association between asthma and obesity, as presented in the international literature [28].

Increasing age independently affected the severity of OSA in our study. This is analogous to the results of Andersen et al. [17]; however, Scott et al. found that age, as a sole independent variable, does not significantly affect OSA severity [24].

In the present study, a predominance of male patients was observed; however, there was no significant correlation between sex and the severity of OSA in either the age or weight groups. In contradiction, according to the international literature, the male sex is an independent risk factor for paediatric OSA [29].

Although, according to the ROC curve, the sensitivity and specificity values, BMI percentile and age can significantly predict severe OSA, the association presents a limited clinical significance. Further research is needed to evaluate possible objective diagnostic parameters, which can help verify severe OSA, in addition to the limitedly available overnight polysomnography. There are promising studies about home sleep apnoea testing; however, the clinical use for diagnosis in the paediatric population remains controversial [30].

Our cohort study revealed that obesity was more prevalent in older paediatric age; however, the strength of the association can be affected by several factors, such as socioeconomic status. For instance, low socioeconomic status can lead to an increased rate of obesity, as compared to higher socioeconomic status, with the correlation of a stagnating or decreased tendency [31].

The present study had some limitations. First, is the limited number of asthmatic patients in our study; additional studies included a larger cohort to explore the role of asthma in OSA. Furthermore, similarly to a previous study [17], the diagnosis of asthma was self-reported and only registered if the patients used asthma medications at the time of the examination. Notwithstanding, lung function testing can provide a more accurate diagnosis. Furthermore, limited information was available on the Brodsky score of adenotonsillar hypertrophy in the patient documentation; thus, adenotonsillar hypertrophy could not be explored as a risk factor. Further limitations can occur when children of varying ages are grouped into a single cohort. For instance, a 3-year-old and a 17-year-old child can be included in the same group during the analysis; however, they had markedly different physiques.

The primary strength of our study is that the cohort was relatively large compared to other studies.

Since the prevalence of obesity is increasing, more children will be affected by OSA and its complications. Our results emphasise an emerging need for early diagnosis and prevention, especially in older age and obesity.

## Conclusions

According to our study, the severity of OSA in a paediatric population depends on body weight status and age. These results indicate that obese, and older children are at increased risk for more severe OSA. However, based on our results, BMI percentile and age have limited clinical relevance in predicting severe OSA. We found a significant association between BMI percentile and OSA severity under 7 years of age, highlighting that in younger children, in addition to adenotonsillar hypertrophy, obesity is an essential risk factor for developing OSA. Further research is required to evaluate the association among excess weight, paediatric bronchial asthma, age, sex, and OSA. Finally, there is an emerging need to create an objective screening methods for paediatric OSA.

## Data Availability

Data will be made available on reasonable request.

## References

[1] Walter LM, Shepherd KL, Yee A, Horne RSC (2020). Insights into the effects of sleep disordered breathing on the brain in infants and children: Imaging and cerebral oxygenation measurements. Sleep Med Rev..

[2] American Thoracic Society (1996). Standards and indications for cardiopulmonary sleep studies in children. Am J Respir Crit Care Med.

[3] Marcus CL, Brooks LJ, Draper KA, Gozal D, Halbower AC, Jones J, Schechter MS, Sheldon SH, Spruyt K, Ward SD, Lehmann C, Shiffman RN, American Academy of Pediatrics (2012). Diagnosis and management of childhood obstructive sleep apnea syndrome. Pediatrics..

[4] Kanney ML, Harford KL, Raol N, Leu RM (2020). Obstructive sleep apnea in pediatric obesity and the effects of sleeve gastrectomy. Semin Pediatr Surg..

[5] Gulotta G, Iannella G, Vicini C, Polimeni A, Greco A, de Vincentiis M, Visconti IC, Meccariello G, Cammaroto G, De Vito A, Gobbi R, Bellini C, Firinu E, Pace A, Colizza A, Pelucchi S, Magliulo G (2019). Risk factors for obstructive sleep apnea syndrome in children: state of the art. Int J Environ Res Public Health.

[6] Kang KT, Chou CH, Weng WC, Lee PL, Hsu WC (2013). Associations between adenotonsillar hypertrophy, age, and obesity in children with obstructive sleep apnea. PLoS One..

[7] Nittari G, Scuri S, Sagaro GG, Petrelli F, Grappasonni I (2020). Epidemiology of obesity in children and adolescents. Teamwork Healthcare.

[8] Graw-Panzer KD, Muzumdar H, Jambhekar S, Goldstein NA, Rao M (2010). Effect of increasing body mass index on obstructive sleep apnea in children. Open Sleep J.

[9] Kang KT, Lee PL, Weng WC, Hsu WC (2012). Body weight status and obstructive sleep apnea in children. Int J Obes (London).

[10] Konstantinopoulou S, Sideris GA, DelRosso LM (2016). The role of co-morbidities. Curr Probl Pediatr Adolesc Health Care.

[11] Wang R, Mihaicuta S, Tiotiu A, Corlateanu A, Ioan IC, Bikov A (2022). Asthma and obstructive sleep apnoea in adults and children—an up-to-date review. Sleep Med Rev..

[12] Benedek P, Lázár Z, Bikov A, Kunos L, Katona G, Horváth I (2013). Exhaled biomarker pattern is altered in children with obstructive sleep apnoea syndrome. Int J Pediatr Otorhinolaryngol.

[13] Alkhalil M, Schulman E, Getsy J (2009). Obstructive sleep apnea syndrome and asthma: what are the links?. J Clin Sleep Med.

[14] Capdevila OS, Kheirandish-Gozal L, Dayyat E, Gozal D (2008). Pediatric obstructive sleep apnea: complications, management, and long-term outcomes. Proc Am Thorac Soc..

[15] Katz ES, D'Ambrosio CM (2010). Pediatric obstructive sleep apnea syndrome. Clin Chest Med..

[16] Barlow SE, Expert Committee (2007). Expert committee recommendations regarding the prevention, assessment, and treatment of child and adolescent overweight and obesity: summary report. Pediatrics..

[17] Andersen IG, Holm JC, Homøe P (2019). Obstructive sleep apnea in children and adolescents with and without obesity. Eur Arch Otorhinolaryngol..

[18] Kaditis AG, Alexopoulos EI, Hatzi F, Karadonta I, Chaidas K, Gourgoulianis K, Zintzaras E, Syrogiannopoulos GA (2008). Adiposity in relation to age as predictor of severity of sleep apnea in children with snoring. Sleep Breath..

[19] Benedek P, Balakrishnan K, Cunningham MJ, Friedman NR, Goudy SL, Ishman SL, Katona G, Kirkham EM, Lam DJ, Leboulanger N, Lee GS, Le Treut C, Mitchell RB, Muntz HR, Musso MF, Parikh SR, Rahbar R, Roy S, Russell J, Sidell DR, Sie KCY, Smith RJ, Soma MA, Wyatt ME, Zalzal G, Zur KB, Boudewyns A (2020). International Pediatric Otolaryngology group (IPOG) consensus on the diagnosis and management of pediatric obstructive sleep apnea (OSA). Int J Pediatr Otorhinolaryngol..

[20] Bitners AC, Arens R (2020). Evaluation and management of children with obstructive sleep apnea syndrome. Lung..

[21] Brouillette RT, Manoukian JJ, Ducharme FM, Oudjhane K, Earle LG, Ladan S, Morielli A (2001). Efficacy of fluticasone nasal spray for pediatric obstructive sleep apnea. J Pediatr..

[22] Sateia MJ (2014). International classification of sleep disorders-third edition: highlights and modifications. Chest..

[23] Mitchell RB, Archer SM, Ishman SL, Rosenfeld RM, Coles S, Finestone SA, Friedman NR, Giordano T, Hildrew DM, Kim TW, Lloyd RM, Parikh SR, Shulman ST, Walner DL, Walsh SA, Nnacheta LC (2019). Clinical practice guideline: tonsillectomy in children (Update). Otolaryngol Head Neck Surg..

[24] Scott B, Johnson RF, Mitchell Md RB (2016). Obstructive sleep apnea: differences between normal-weight, overweight, obese, and morbidly obese children. Otolaryngol Head Neck Surg..

[25] Xu Z, Jiaqing A, Yuchuan L, Shen K (2008). A case-control study of obstructive sleep apnea-hypopnea syndrome in obese and nonobese chinese children. Chest..

[26] Narayanan A, Yogesh A, Mitchell RB, Johnson RF (2020). Asthma and obesity as predictors of severe obstructive sleep apnea in an adolescent pediatric population. Laryngoscope.

[27] Julien JY, Martin JG, Ernst P, Olivenstein R, Hamid Q, Lemière C, Pepe C, Naor N, Olha A, Kimoff RJ (2009). Prevalence of obstructive sleep apnea-hypopnea in severe versus moderate asthma. J Allergy Clin Immunol.

[28] Peroni DG, Pietrobelli A, Boner AL (2010). Asthma and obesity in childhood: on the road ahead. Int J Obes (Lond).

[29] Wang J, Zhao Y, Yang W, Shen T, Xue P, Yan X, Chen D, Qiao Y, Chen M, Ren R, Ren J, Xu Y, Zheng Y, Zou J, Tang X (2019). Correlations between obstructive sleep apnea and adenotonsillar hypertrophy in children of different weight status. Sci Rep.

[30] Kirk V, Baughn J, D'Andrea L, Friedman N, Galion A, Garetz S, Hassan F, Wrede J, Harrod CG, Malhotra RK (2017). American academy of sleep medicine position paper for the use of a home sleep apnea test for the diagnosis of OSA in children. J Clin Sleep Med.

[31] Hemmingsson E (2018). Early childhood obesity risk factors: socioeconomic adversity, family dysfunction, offspring distress, and junk food self-medication. Curr Obes Rep.

